# Application of Polyurethane Foam as a Material for Reducing Vibration of Wheelchair User

**DOI:** 10.3390/ma18061280

**Published:** 2025-03-14

**Authors:** Gabriela Chwalik-Pilszyk, David Cirkl, Marek S. Kozien

**Affiliations:** 1 Faculty of Mechanical Engineering, Cracow University of Technology, 31864 Cracow, Poland; marek.kozien@pk.edu.pl; 2 Faculty of Mechanical Engineering, Technical University of Liberec, 46117 Liberec, Czech Republic; david.cirkl@tul.cz

**Keywords:** polyurethane foam, vibroisolation, multibody system

## Abstract

In this article, an attempt was made to model the body of a person moving in a passive manner (movement forced by another person) in a wheelchair. For this purpose, the Wan–Schimmels model was modified by 4 DOF, supplementing it with the weight of the wheelchair and a polyurethane cushion. The study was designed to test the effectiveness of utilizing a polyurethane cushion to reduce the whole-body vibration acting on a person while moving in a wheelchair. The study used a rheological model of polyurethane (PU) foam with concentrated parameters. Harmonic and random vibration analysis was carried out for this model. At the same time, the model with 5 DOF seems to be sufficient to describe vibrations transmitted to wheelchair users. The model presented in this paper can become a tool for future analysis of vibrations of people of different weights, moving passively on various types of wheelchairs on surfaces whose irregularities can be given by an appropriate form of kinematic excitation. The approach used in this study is likely to be useful in selecting a wheelchair and seat cushion so as to counteract and minimize vibrations perceived by humans.

## 1. Introduction

Manual wheelchairs largely determine the possibilities of adapting to life for people with permanent mobility disabilities. However, when moving in a wheelchair, a disabled person is exposed to whole-body vibrations. Studies show that constant exposure to vibration can lead to poor body posture, with the consequence that the wheelchair user is more susceptible to additional injuries [1,2,3,4]. The amount of vibration received also depends on the type of construction and additional equipment for the wheelchair, such as shock absorbers or seat cushions. However, this equipment does not sufficiently dampen vibrations, which is confirmed by tests [5,6,7]. The harmful effects of whole-body vibrations on the spine have been observed. The most commonly reported adverse reactions resulting from the effects of vibrations are low back pain and lumbar disc hernia [8,9,10].

In the literature, there are some experimental studies on the impact of vibrations during wheelchair riding [5,9,11]. The author made some experimental investigations in estimating the influence of passive wheelchair motion on the vibration of a disabled person and the influence of different types of road pavement on the amplitudes of human body vibrations [12]. Relatively small differences in vibration characteristics of vehicles (not only wheelchairs) may have a great influence on driver and passenger comfort [13].

Vibroisolation of seats to minimize the vibrations of the human body is a commonly applied process to prevent transmission of vibration from its base (usually of a kinematic type) to the human body [14]. It is commonly used in the design of passenger and driver seats in cars, buses, trains, and airplanes—see, e.g., [15]. It is realized among passive, semi-active, active, and hybrid systems of vibroisolation [16]. It should be pointed out that the passive systems’ vibration reduction can be applied to coupled fields. An example of this is the system of reduction in plate structural vibrations (solid body) by piezoelectric elements (electric field) to minimize sound radiation (acoustic field). An interesting type of vehicle is the wheelchair [1,4]. On the one side, its construction should be simple and lightweight to make it a user-friendly system of car/train transport and home storage. On the other side, it should be a source of transmission of vibrations coming from motion on not-plane surfaces of roads and street pavements to the body of a disabled person. There is a limited number of texts dealing with the problem of vibration reduction in people using wheelchairs.

Different materials with viscoelastic properties are designed and used to reduce the vibration of different structures. As an example, it can be elements with polylactide [17], functionally graded porous structures [18], or a viscoelastic core layer [19].

In order to properly analyze the issues of the impact of vibrations on the human body and design vibration isolation, it is necessary to conduct frequency analyses of signals [16,20]. Fourier analysis is most often used for this purpose [21].

The research focuses on identifying the material that acts as a vibration isolator and is placed between the seat of the wheelchair and the person sitting on it in place of the commonly used air springs for seats. Due to its usefulness, technology process, health neutrality, ease of cleaning, and low price, polyurethane material has been chosen for application. The general idea was applied to car seats—e.g., [22,23]. The main problem with the design of a vibroisolator made of polyurethane foam is the identification of material properties and their strong dependence on density (manufacturing process) and frequency (dynamic type of application) [24,25,26,27,28].

There are several models of the human body in the sitting position in the literature [29,30,31]; however, the model presented by Weerapong and co-authors is the only one that relates to a person in a wheelchair [32,33,34,35]. It seems crucial to create a universal model of the human body to simulate vibrations using different types of wheelchairs. In research, linear models are most often applied [31,32,36,37]. Non-linear models are much less common in applications [29]. The most popular models found in the literature are the Wan and Schimmels model [31] and the Boileau and Rakheja model [36] and modifications of these models [37]. A wide variety of models used can be found in the literature. Among discrete models, those ranging from one to a dozen degrees of freedom are utilized [37,38].

Studies show that a higher number of degrees of freedom of models does not necessarily provide better matching accuracy compared to simple models [29]. In addition, models with four degrees of freedom have a sufficient number of parameters and provide a good enough match to experimental data [29]. Therefore, this work presents the use of a modification of the model with 4 DOF to describe a person in a wheelchair. The models presented in the literature do not provide a universal solution for describing the biodynamic response of the human body in any situation (while driving a car, etc.). Therefore, it seems important to create this type of model (human–wheelchair), which would provide information about the dynamic response of the human body. This will reduce the necessity to perform experimental tests and thus expose people with disabilities to additional vibrations (e.g., in impulse vibration analyses).

This paper presents the application of a mathematical model to study the mechanical response of the wheelchair–human system. The model includes the use of a polyurethane cushion on the wheelchair seat to reduce vibration. The analysis used a lumped parameters model, which is one of the most commonly used model types in biomechanics [6]. Lumped parameters models treat humans as rigid bodies connected to each other by an elastic-damping element. The advantage of these types of models is that they are simple to analyze and easy to validate with experimental results. The drawback, however, is the often-stated limitation to unidirectional analysis [39]. This analysis focuses on vibrations in the vertical direction because they are the most dangerous from the point of view of human body response.

## 2. Polyurethane Foam and Its Material Properties

Polyurethane foam arises during a vigorous reaction of chemical agents when carbon dioxide is released, which is simultaneously accompanied by the solidification of a material matrix in a form that gives the final shape to the product. The result of this process is a material with a cell structure either with closed or opened pores. For applications in the field of sitting comfort, the use of foams with opened cells became very widespread. From the point of view of statistics, the size of the cells and the length of the cell’s struts are random variables. As shown in [40], the distribution of these structural features is skewed toward a higher frequency of smaller sizes within the material volume.

The polyurethane foam in seating applications influences the comfort of sitting and traveling because, from a dynamic point of view, it dampens shocks and vibrations caused by road surface irregularities transmitted to the sitting person. From a static point of view, it also influences the distribution of pressure in the contact zone between the seat cushioning and the sitting person. All these phenomena are influenced by the mechanical properties of foam, which is considered a non-linear viscoelastic material. The use of foam materials is usually found in applications with mostly compressional loads.

**Figure 1:**
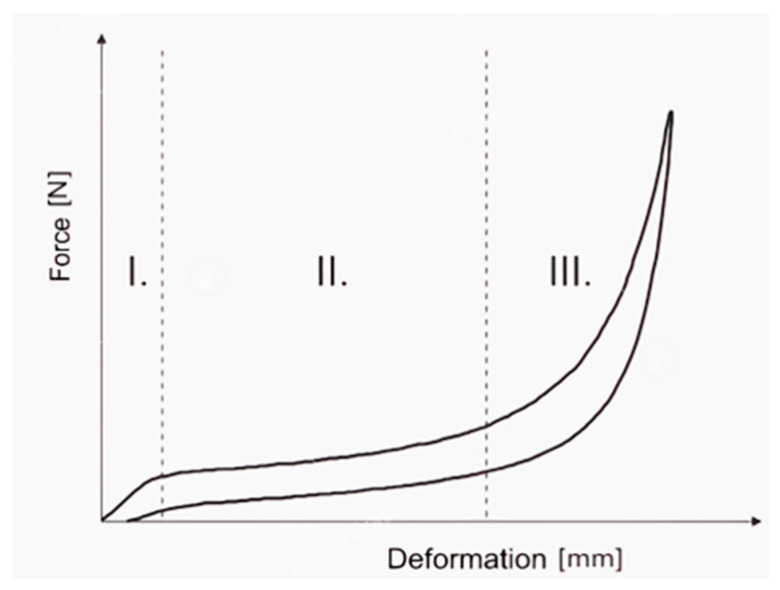
Typical three-phase force response of PU foam specimen to compression load.

In the case of uniaxial compressional loads, the overall non-linear force response shows a typical hysteresis character, as depicted in Figure 1. It is possible to divide it into three phases. The first phase is characterized by an almost linear force increase. When we consider a cell as a fundamental structure element, at this phase, the buckling strength of the cells has not been overcome yet. With increasing deformation, phase II follows, characterized by considerably lower and again almost linear force increase. Because of its nearly flat character, it is usually called a plateau. In this interval, some cells have already collapsed, with very high local deformation, while others still resist due to their higher buckling strength. For foams with lower densities, a high non-homogeneous distribution of local deformation appears. The structure locally collapses in areas whose propagation is related to the topological distribution of cells with lower buckling strength. Phase III starts with the upcoming homogenization of local deformation when most cells have already collapsed. This phase is typical of material densification by pressing out the air from the structure volume. Foam loses properties given by the character of solid structure, and its stiffness tends to approach values that a non-foamed polymeric material would have.

The article [41] deals with the measurement and recognition of heterogeneous deformation fields appearing during compression. From that, it follows that the most important factor influencing stiffness in phase II is material density. Foams with lower density show a tendency for more heterogeneous local deformation during phases I and II. This practically leads to a more distinct plateau phase, as local deformations become more homogenous. Conversely, in foams with higher density, the plateau phase tends to disappear. This is because the material structure is more compact, reducing the occurrence of localized deformation zones. The force–displacement curve then exhibits a more progressive character across all compression phases, with a smoother and more continuous increase in force.

**Figure 2:**
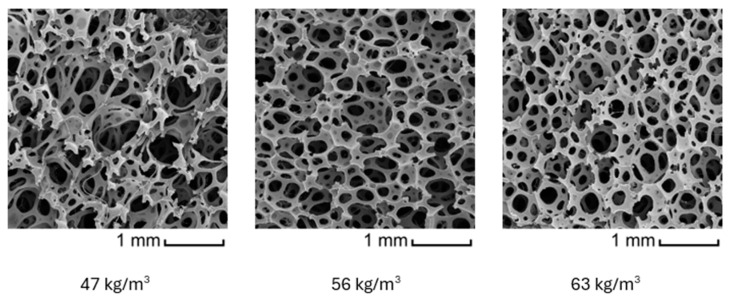
SEM images of foam structures of different material density.

The illustrative pictures made by a scanning electron microscope (SEM) in Figure 2 show the difference in the cell structure of foam specimens made from foam material of the same chemical formulation. These specimens only differ by material density.

The total force response of foam to compressional deformation is generally assumed to consist of two main components: a restoring force and a damping force. The restoring force represents the elastic properties of the foam structure as it attempts to return to its original shape after compression. Meanwhile, the damping force is a manifestation of energy dissipation mechanisms that occur during deformation.

Damping itself is not a single phenomenon but rather a combination of different effects. One major contributor is the internal damping of the polyurethane material matrix, which occurs when the thin struts forming the foam’s cellular structure bend and deform. It is assumed to play a more significant role at lower relative deformations (phases I and II).

As compression increases and the material enters higher deformation phases (phases II and III), another damping mechanism can be taken into consideration: frictional interactions between the foam’s cell struts. When these struts come into contact, additional energy can be dissipated through friction. This effect becomes particularly pronounced as the foam structure progressively collapses and densifies.

Another type of energy dissipation might be connected with aerodynamic choking when air contained in foam cells is pressed out during compression and sucked back during unloading. Changing the cells’ dimensions during deformation acts as changing the size of choking openings with variable cross-sections. The influence of this phenomenon on total foam damping was investigated experimentally in [42]. A comparison of the measured force response of foam material in atmospheric and vacuum conditions showed that the contribution of aerodynamic choking to total damping is very low and unmeasurable for common rates of deformation.

The typical effect of damping is a phenomenon of stress relaxation. This effect is very significant in the case of flexible structured materials like foams. It was proven through experimental measurements that common foams used in seating applications lose up to 30% of their force response value to the step compression load within several minutes. In the case of special materials, such as low spring back foams with extremely high damping originally designed for special seating applications, the relaxation effect causes about a 60% force decrease. Another significant effect of damping is an increase in material stiffness for higher rates of deformation during the loading process, which leads to a higher amount of dissipated energy.

## 3. Wan–Schimmels Model

**Figure 3:**
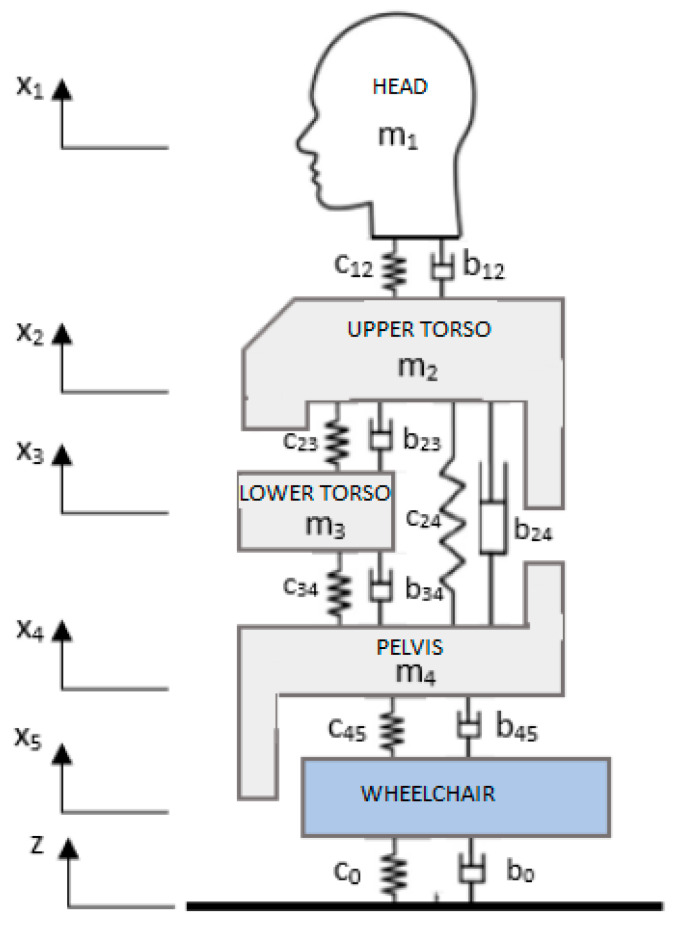
Wan–Schimmels model with a wheelchair body.

The Wan–Schimmels model was used to represent the dynamic response of the body of a human moving in a wheelchair, supplementing it with an additional mass modeling the entire wheelchair (Figure 3). The final result is a five-degree-of-freedom model consisting of four segments of the human body: head (1), upper torso (2), lower torso (internal organs) (3), pelvis with lower limbs (4), and wheelchair (5). In this model, the human body is in a sitting position. The lower part of the body is supported on the seat of the wheelchair, while the upper segments remain unsupported. In this model, the external excitation was treated as the kinematic excitation of the wheelchair from its movement on uneven surfaces and transmitted in the form of vibrations to the human body through the seat.

The individual segments are connected by linear springs and linear dampers (the Voigt–Kelvin model), whose stiffness coefficients and damping coefficients are marked as *c_ij_* and *b_ij_*, respectively, where i or j identifies the number of the mass and its displacements (i, j = 1, 2, …, 5), and c_0_ and b_0_ stand for the coefficients associated with the ground. The system of five ordinary differential equations, describing the vibrations of the elements of the built model (with five degrees of freedom), has the Formula (1).

(1) $$\left\{\begin{array}{l} \begin{array}{l} m_{1}{\ddot{x}}_{1}=-b_{12}\left({\dot{x}}_{1}-{\dot{x}}_{2}\right)-c_{12}(x_{1}-x_{2})\\ m_{2}{\ddot{x}}_{2}=b_{12}\left({\dot{x}}_{1}-{\dot{x}}_{2}\right)+c_{12}\left(x_{1}-x_{2}\right)-b_{23}\left({\dot{x}}_{2}-{\dot{x}}_{3}\right)-c_{23}\left(x_{2}-x_{3}\right)-b_{24}\left({\dot{x}}_{2}-{\dot{x}}_{4}\right)-c_{24}(x_{2}-x_{4}) \end{array}\\ \begin{array}{l} m_{3}{\ddot{x}}_{3}=b_{23}\left({\dot{x}}_{2}-{\dot{x}}_{3}\right)+c_{23}\left(x_{2}-x_{3}\right)-b_{34}\left({\dot{x}}_{3}-{\dot{x}}_{4}\right)-c_{34}\left(x_{3}-x_{4}\right)\\ m_{4}{\ddot{x}}_{4}=b_{34}\left({\dot{x}}_{3}-{\dot{x}}_{4}\right)+c_{34}\left(x_{3}-x_{4}\right)+b_{24}\left({\dot{x}}_{2}-{\dot{x}}_{4}\right)+c_{24}(x_{2}-x_{4})-b_{45}\left({\dot{x}}_{4}-{\dot{x}}_{5}\right)-c_{45}(x_{4}-x_{5})\\ m_{5}{\ddot{x}}_{5}=b_{45}\left({\dot{x}}_{4}-{\dot{x}}_{5}\right)+c_{45}(x_{4}-x_{5})-b_{0}\left({\dot{x}}_{5}-\dot{z}\right)-c_{0}(x_{5}-z) \end{array} \end{array}\right.$$

The values of the spring and the damping coefficients were taken in accordance with the values given in the articles [43,44]. On the other hand, the model’s weight was approximately in line with that of the human participant in the experimental study published by Chwalik and co-authors [45]. The mass value m_5_ was taken as the mass of the Unix Breezy Sunrix Medical wheelchair used in the experimental study. The *c_o_* and *b_o_* coefficients were determined on the basis of custom tests performed in the laboratory. A summary of the assumed model values is given collectively in Table 1.

## 4. Simulation of Human Body Response

**Figure 4:**
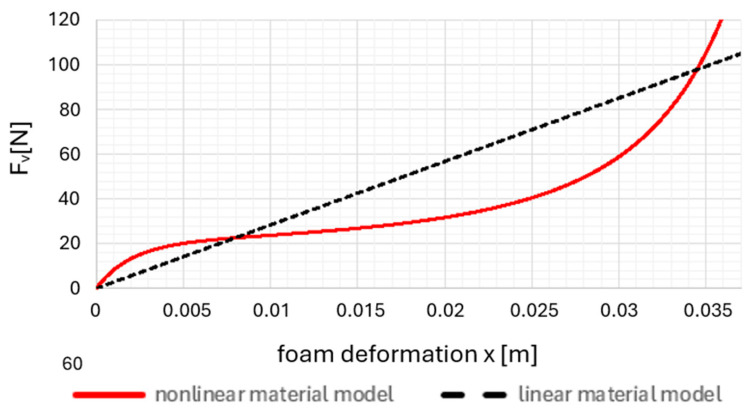
The force curve of a linear and nonlinear material model.

The stated non-linear restoring force characteristic of PU foam was adjusted with a straight line to obtain stiffness *k* for a linear material model. In this case, it is not a linearization of the nonlinear restoring force of PU foam (Figure 4). Therefore, the stiffness constant for the system with a linear material model c = 2837 Nm^−1^ and the damping constant *b* = 30 N∙sm^−1^ were chosen. These values were included in the c_45_ and b_45_ coefficients to simulate the polyurethane cushion on the wheelchair seat.

**Figure 5:**
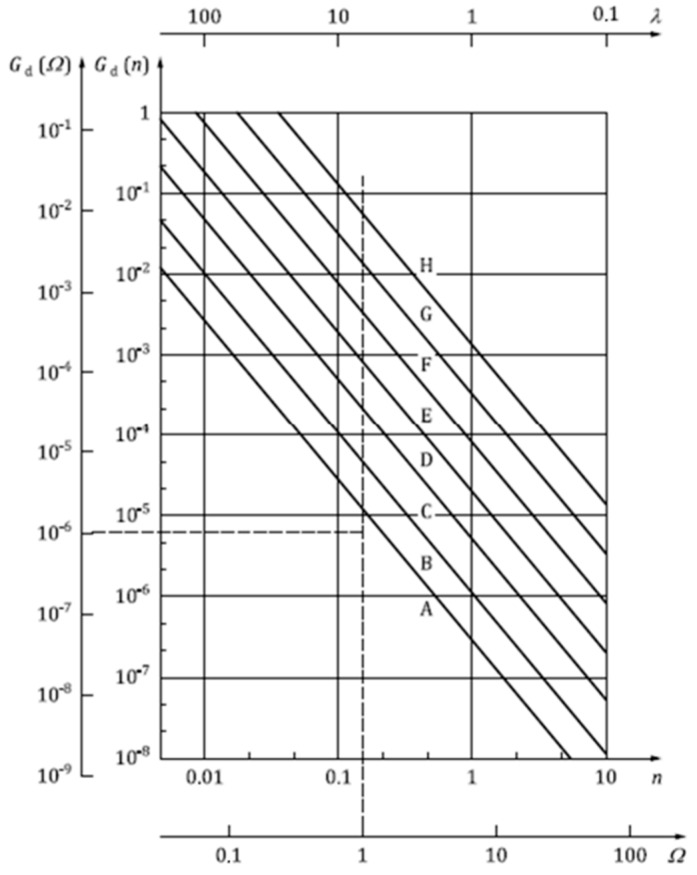
Classification of roads according to the criterion of spectral density of displacements (road irregularities) (ISO 8608, 2016) [46].

For the presented model, the analysis was carried out assuming that the form of the kinematic excitation function describing the path irregularity has the character of a random variable. The simulation used the normalized forms of displacement power spectral density (road irregularities) contained in ISO 8608:2016(E) [46] and classifying the surface on which a wheelchair travels as a road between categories B and C, E and F, or G and H (Figure 5). For the simulations, roads in categories B and C and E and F were selected, for which the excitation spectral density for specific frequencies was determined based on the ISO standard.

Standard ISO 8608 [46] specifies the road classification of random road profiles based on vertical displacement power spectral density (PSD). ISO 8608 does not specify a correlation between road class and road category or road quality [36].

For the presented model, an analysis was also carried out for a kinematic excitation in the form of vibrations generated when a wheelchair moves over uneven roads. For this purpose, the signal obtained from the experimental studies presented in the paper [12] was used. For the simulation, the time waveform obtained during a ride on a hexagonal cube was used, and the measurement was performed on the seat of the wheelchair [12].

Since in the discrete model (Figure 3), the external excitation is of a kinematic excitation type describing the unevenness of the ground (road), it became necessary to estimate the course of the displacement power spectral density function or the acceleration power spectral density of this function based on the knowledge of the acceleration power spectral density function measured on the seat.

For stationary waveforms of the excitation function, the relation between the spectral density functions of the excitation *G_z_(ω)* and the response *G_x_(ω)* can be given in the form (2) [34], where the spectral bandwidth function *H(ω)* with reference to the solution function (3) has the Formula (4). The correlation (5) between the displacement spectral density function *G_x_(ω)* and the acceleration spectral density function *S_a_(ω)* is fulfilled.

(2) $$G_{x}\left(\omega \right)={\left|H\left(\omega \right)\right|}^{2}\;G_{z}(\omega ),$$

(3) $$x\left(t\right)=\sqrt{\frac{1+{\left(2\gamma \mu \right)}^{2}}{{\left(1-\mu^{2}\right)}^{2}+{\left(2\gamma \mu \right)}^{2}}}\sin\left(\omega t-\varphi \right)=x_{0}\sin\left(\omega t-\varphi \right),$$

(4) $$H\left(\omega \right)=\frac{x_{0}}{z_{0}}=\sqrt{\frac{1+{\left(2\gamma \mu \right)}^{2}}{{\left(1-\mu^{2}\right)}^{2}+{\left(2\gamma \mu \right)}^{2}}}$$

(5) $$G_{a}\left(\omega \right)=\omega^{4}{\;G}_{x}\left(\omega \right)$$

Based on the above formulas, the approximate waveforms of the spectral density function of the surface kinematic excitation acceleration were determined on the basis of the results obtained in the experimental tests (a run on a hexagonal slab-type pavement) presented in the publication [12]. The discretized values of the function, in the frequency range 1–4 Hz, analyzed further and applied in further simulations, are given in Table 2.

Analysis of induced vibrations using the fem Ansys package was carried out using the transition state dynamic analysis module (ANTYPE,SPECTR) with the PSD option. The excitation of driving on a hexagonal slab was assumed in the form of discrete values of the spectral density of surface displacements *G_z_(f)* given in Table 2 and based on the ISO standard. The acceleration dispersion for individual nodes/masses was calculated based on Equation (6).

(6) $$\sigma_{x}^{2}=\frac{1}{2\pi }\int_{0}^{+\infty }G_{x}(\omega )\;d\omega$$

Table 3 and Table 4 summarize the displacement and velocity dispersion values obtained in the simulations for individual elements (masses, nodes) under limit curves for road categories B and C and E and F according to ISO 8608:2016(E) [46].

Table 5 compares the values of displacement and acceleration dispersion for simulations using the time course obtained in experimental studies [12].

This paper presents the use of the road profiles included in ISO 8608 as a kinematic excitation acting on a wheelchair user. Currently, this classification is used for simulation purposes to solve various tasks in vibration analysis of mechanical and building structures and in the road-vehicle-driver interaction system [32]. There are no studies in the literature that have applied this standard to simulate the driving of a disabled person in a wheelchair.

The obtained values of displacement and acceleration dispersion in the conducted driving simulations, on roads of various categories according to ISO 8608, seem to be plausible. In an experimental study by Duvall et al. [47], the value of vibrations received on the seat of a wheelchair ranges from 0.5 to 5.4 m/s^2^, depending on the surface on which the wheelchair was driven. In contrast, Hashizume et al. [48] found that the value varies between 1 and 3 m/s^2^. However, in the publication by Hischke and co-publishers, these values are in the range of 1.2–1.4 m/s^2^ [49]

These values are close to those obtained in the simulations. Yet, the available experimental studies lack information on the magnitude of perceived vibrations on the head when moving on different types of surfaces.

In the simulation, higher acceleration and displacement values were obtained for the E/F road category. It is also worth noting that ISO 8608 lacks descriptive commentary on the type and condition of roads in a given class. According to Múčka [32], classes B and C may be suitable for simulating low-quality paved surfaces. Category A is a road in good condition [32]. In contrast, road grades D to H often correspond to unpaved roads. In the literature, the ISO 8608 standard is mainly used to account for passenger car safety and comfort thresholds, and for such cases, the recommended maximum vehicle speeds are >100 km/h for class A and B, 30–60 km/h for class C, and <15 km/h for class D [32]. When ISO 8608 is applied to wheelchair passage, the speed assumed in the simulation is 1 m/s. In this paper, it was decided not to carry out simulations for road categories above G. According to the description available in the literature, it can be presumed that roads above class G are roads in poor technical condition, on which it would be impossible to drive a wheelchair at the determined speed.

There are a few models in the literature that describe the human–wheelchair system. However, neither of these models includes an analysis of random vibrations on the human body while moving in a wheelchair and therefore does not represent movement on the various types of surfaces found in public spaces.

Weerapong and co-authors proposed a model of a wheelchair and a passenger in an unsupported sitting position with 9 DOF [33,50] and 11 DOF [34,35]. In these models, the simulation is carried out for sinusoidal excitation. For a model with 11 degrees of freedom, they studied the response in the system in the frequency range of 1–15 Hz [35]. The values obtained range from 1.5 to 2.3 m/s^2^.

The results obtained in the simulation with the amplitudes obtained in the experimental tests (Table 5) are similar to the values obtained for the B and C road categories (Table 4). This approach will allow simulating real runs on different types of surfaces on which a wheelchair user moves.

Based on the results obtained in the simulation, the lowest acceleration value was observed for mass 4, that is, for the pelvis with lower limbs. A similar dependence can be seen in experimental studies. In the study by Weerapong and co-survivors [35], the torso was the most responsive body segment, while the pelvis was the least responsive. In addition, they observed that a wheelchair seat cushion was effective in reducing vibration levels in all body segments. The use of the cushion brought the vibrations below the ISO exposure limit curve for a 25 min exposure.

**Figure 6:**
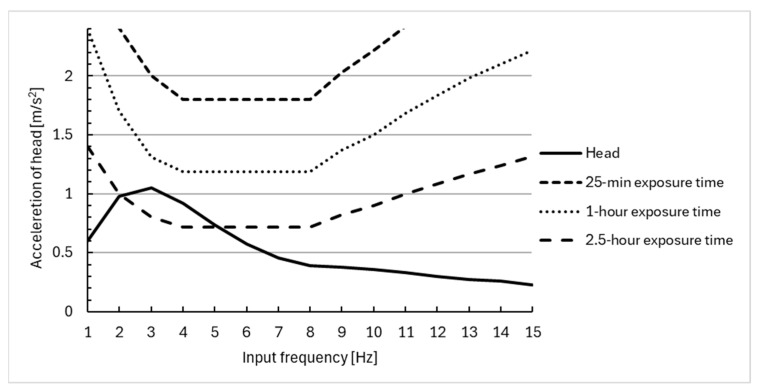
Comparison of vibration acceleration at the head of wheelchair users with a sinusoidal input at different frequencies, using the ISO 2631 [51] standards for human exposure to vibration.

**Figure 7:**
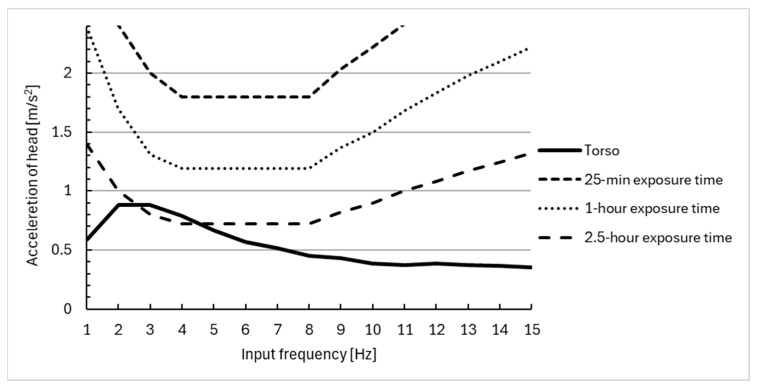
Comparison of vibration acceleration at the torso of wheelchair users with a sinusoidal input at different frequencies, using the ISO 2631 [51] standards for human exposure to vibration.

**Figure 8:**
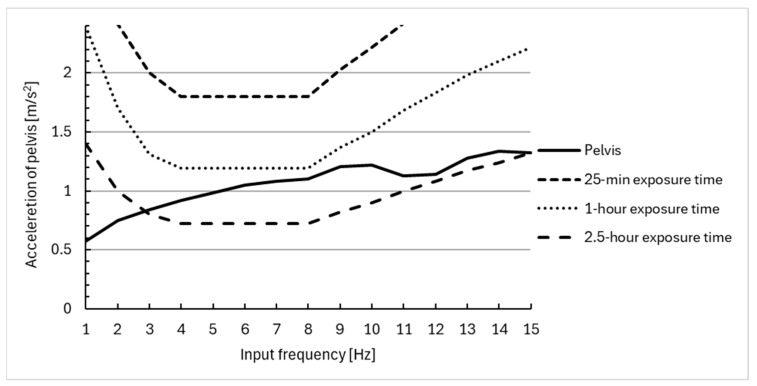
Comparison of vibration acceleration at the pelvis of wheelchair users with a sinusoidal input at different frequencies, using the ISO 2631 [51] standards for human exposure to vibration.

The response of the human body model to harmonic excitation with frequencies in the range of 1–15 Hz is shown in Figure 6, Figure 7 and Figure 8. In addition, the 25 min, 1 h, and 1.5 h exposure limit curves defined by ISO 2631 [51] are superimposed in Figure 6, Figure 7 and Figure 8. This enabled the evaluation of the effectiveness of vibration reduction by the polyurethane cushion.

The maximum acceleration value for the head equal to 1.05 m/s^2^ occurs at an excitation frequency of about 3 Hz. For the torso, on the other hand, the value is equal to 0.78 m/s^2^ for an excitation frequency of 2 Hz.

The results suggest that the use of a polyurethane seat cushion on a wheelchair can effectively reduce the acceleration of all segments of the model. The acceleration values obtained do not exceed those for a 1 or 2.5 h exposure to vibration, and therefore, the seat cushion can improve the comfort of using a wheelchair. The results obtained in this paper are lower than the values obtained in the experimental studies conducted by the authors and presented in [45]. With harmonic forcing at a frequency of 2 [Hz], the values obtained on the human head are within the range of 1.5–2 m/s^2^, and therefore, there is an evident decrease in the magnitude of the received vibrations due to the seat cushion used.

The 5 DOF model presented in this paper seems to be sufficient to describe the vibrations transmitted to wheelchair users. At the same time, knowing the spring and damping properties of seat cushions, it is possible to perform simulations for any material to study the damping capacity and reduce the amount of transmission of harmful vibrations to a person moving in a wheelchair. The study focuses on the initial state case responses of various parts of the body; a study of transient responses would also be valuable. Vibrations that arise, for example, when passing curbs or other obstacles, often generate a higher acceleration and may have a different effect on the human body than initial state case vibrations.

The presented model can be used as a tool for future vibration analysis of people of different weights moving passively in a wheelchair on various surfaces. Pavement irregularities can be determined, for example, by determining the power spectral density of displacement or acceleration, either using data from measurements or specified in standards. The model makes it possible to simulate driving on various types of wheelchairs, if their stiffness and damping coefficients are known. The model is used for unidirectional linear analysis (vertical vibrations). For whole-body vibrations acting on the human body, transverse vibrations are also relevant. In the future, it would be advisable to consider a model in which transverse vibration analysis would also be possible.

## 5. Conclusions

This study showed the possibility of using a modification of the Wan–Schimmel model to describe the wheelchair–human system. This solution seems to be universal for different types of wheelchairs if we know in advance (determine) the values of stiffness and damping parameters.

The research presented here demonstrates the feasibility of simulations using various types of seat cushions to determine their damping capacity. The approach used in this study is likely to be useful in selecting a wheelchair and seat cushion so as to counteract and minimize vibrations perceived by humans.

The analysis of random vibrations for a stationary excitation is shown, in which it is possible to determine the dispersion of displacements using analytically determined relevant spectral bandwidth functions H(ω) and then transmittance functions. This modeling approach facilitates the ability to conduct analyses of a qualitative and quantitative nature. The approach also allows simulations to be carried out for different values of paths, whose inequalities can be defined, for example, by determining the spectral power density of the displacement or acceleration.

The next step in modeling the problem should be to conduct the transient analysis. This type of analysis allows for taking into account the nonlinear characteristic of polyurethane (see Figure 1) as well as the pulse-type excitation (e.g., resulting from the wheelchair hitting the curb). Taking into account the assessment of the impact of vibrations on the wheelchair user’s body, it is worth considering the jerk parameter in the case of pulse-type loads in addition to the commonly used parameter of acceleration.

## Figures and Tables

**Table 1:** Biodynamic model parameter values.

Mass (m_i_) [kg]	Spring Constant (c_ij_) [Nm^−1^]	Damping Constant (b_ij_) [Nsm^−1^]
m_1_ = 4.17	c_12_ = 134,400	b_12_ = 250
m_2_ = 15	c_23_ = 10,000	b_23_ = 200
m_3_ = 5.5	c_24_ = 192,000	b_24_ = 909.1
m_4_ = 36	c_34_ = 20,000	b_34_ = 330
m_5_ = 18	_C45_ = 49,341.6 ^1^	b_45_ = 2475 ^1^

^1^ The value of the coefficients according to the values given in articles [45,46].

**Table 2:** Biodynamic model parameter values determined through experimental studies.

f [Hz]	1	2	4
*G_a_* [m^2^s^−4^Hz^−1^]	4.0 × 10^−9^	5.0 × 10^−8^	5.0 × 10^−6^
${\;G}_{\ddot{z}}$ [m^2^s^−4^Hz^−1^]	3.4 × 10^−9^	2.4 × 10^−8^	5.6 × 10^−7^
*G_z_* [m^2^Hz^−1^]	2.2 × 10^−12^	9.6 × 10^−13^	1.4 × 10^−12^

**Table 3:** Summary of displacement dispersion values when simulating driving on roads of various categories according to ISO 8608.

Number	Element	Displacement Dispersion σ_xi_ [mm]	
		Category B/C	Category E/F
1	Head	1.30	7.47
2	Upper torso	1.29	7.40
3	Lower torso	1.33	7.67
4	Pelvis	1.24	7.14
5	Wheelchair	0.74	4.22

**Table 4:** Summary of acceleration dispersion values when simulating driving on roads of various categories according to ISO 8608.

Number	Element	Acceleration Dispersion σ_xi_ [m/s^2^]	
		Category B/C	Category E/F
1	Head	0.57	3.68
2	Upper torso	0.57	3.63
3	Lower torso	0.57	3.58
4	Pelvis	0.55	3.49
5	Wheelchair	0.31	1.97

**Table 5:** Comparison of the displacement and acceleration dispersion values for simulations.

Number	Element	Dispersion	
		Displacements σ_xi_ [mm]	Accelerations σ_xi_ [m/s^2^]
1	Head	1.59	0.613
2	Upper torso	1.57	0.607
3	Lower torso	1.55	0.630
4	Pelvis	1.51	0.590
5	Wheelchair	0.89	0.340

## Data Availability

The original contributions presented in the study are included in the article, further inquiries can be directed to the corresponding author.
